# The Relationship between Seven Common Polymorphisms from Five DNA Repair Genes and the Risk for Breast Cancer in Northern Chinese Women

**DOI:** 10.1371/journal.pone.0092083

**Published:** 2014-03-18

**Authors:** Peijian Ding, Yang Yang, Luyang Cheng, Xuejun Zhang, Limin Cheng, Caizhen Li, Jianhui Cai

**Affiliations:** 1 Department of Surgery, Hebei Medical University, Shijiazhuang, Hebei, China; 2 Department of Surgery, Hebei General Hospital, Shijiazhuang, Hebei, China; 3 Department of Oncology & Immunotherapy, Hebei General Hospital, Shijiazhuang, Hebei, China; 4 Department of Surgery, The Affiliated Hospital of Chengde Medical College, Chengde, Hebei, China; 5 Hebei Eracon Bio-tech Co. Ltd, Shijiazhuang, Hebei, China; Centro di Riferimento Oncologico, IRCCS National Cancer Institute, Italy

## Abstract

**Background:**

Converging evidence supports the central role of DNA damage in progression to breast cancer. We therefore in this study aimed to assess the potential interactions of seven common polymorphisms from five DNA repair genes (XRCC1, XRCC2, XRCC3, XPA and APEX1) in association with breast cancer among Han Chinese women.

**Methodology/Principal Findings:**

This was a case-control study involving 606 patients diagnosed with sporadic breast cancer and 633 age- and ethnicity-matched cancer-free controls. The polymerase chain reaction - ligase detection reaction method was used to determine genotypes. All seven polymorphisms were in accordance with Hardy-Weinberg equilibrium in controls. Differences in the genotypes and alleles of XRCC1 gene rs25487 and XPA gene rs1800975 were statistically significant between patients and controls, even after the Bonferroni correction (P<0.05/7). Accordingly, the risk for breast cancer was remarkably increased for rs25487 (OR = 1.28; 95% CI: 1.07–1.51; P = 0.006), but decreased for rs1800975 (OR = 0.77; 95% CI: 0.67–0.90; P = 0.001) under an additive model at a Bonferroni corrected alpha of 0.05/7. Allele combination analysis showed higher frequencies of the most common combination C-G-G-C-G-G-G (alleles in order of rs1799782, rs25487, rs3218536, rs861539, rs1800975, rs1760944 and rs1130409) in controls than in patients (P_Sim_ = 0.002). In further interaction analysis, two-locus model including rs1800975 and rs25487 was deemed as the overall best model with the maximal testing accuracy of 0.654 and the cross-validation consistency of 10 out of 10 (P = 0.001).

**Conclusion:**

Our findings provide clear evidence that XRCC1 gene rs25487 and XPA gene rs1800975 might exert both independent and interactive effects on the development of breast cancer among northern Chinese women.

## Introduction

Breast cancer is the most common invasive cancer in women, and like other forms of cancer it results from multiple hereditary and environmental modulators, possibly in an interactive manner. Many risk factors such as ionizing radiation and alcohol consumption have been established to account for approximately 30% of breast cancer patients [1]. Family studies found that the risk for those with first-degree relatives of affected individuals is more than two times higher than the risk of general population [2], [3], confirming a strong genetic component underlying the etiology of breast cancer [4]. Pasche et al have written an excellent review on the genetic underpinnings of breast cancer [5]; however, to determine many genes and which genetic determinants are actually involved in the pathogenesis of breast cancer remains an interpretive challenge.

Evidence is converging supporting the central role of DNA damage in progression to breast cancer. In fact, exposure to ionizing radiation, which can cause double-strand DNA breaks, increased the risk of developing breast cancer [6], [7]. *In-vitro* studies also observed that radiation-induced damage can remarkably reduce the repair proficiency of DNA double-stranded breaks in breast cancer patients [8]. As such, it is reasonable to hypothesize that deficiency in DNA repair proteins induced by genetic mutations can initiate or aggravate the development of breast cancer. However, there is a general impression that most published studies assessing the relationship between DNA repair genes and breast cancer risk have often focused on a single gene or a single polymorphism, but overlooked the potential gene-to-gene interactions, a ubiquitous phenomenon in human genetics. Accordingly, we conducted the present study to assess the genetic interactions of seven common polymorphisms from five DNA repair genes in association with breast cancer among Han Chinese women.

## Materials and Methods

### Study participants

In total, 1239 study participants were enrolled on a hospital-based design from Chengde city, Hebei province, China. Approval of this study was obtained from the Ethics Committee of Chengde Medical College, and each participant read and signed the informed consent before entering this study, which was carried out according to the principles of the Declaration of Helsinki.

All breast cancer patients who had no prior history of any cancers and reported no family history of breast cancer were for their first time diagnosed as invasive ductal carcinoma based on pathological confirmation, and then they received surgical intervention plus adjuvant chemotherapy at the Affiliated Hospital of Chengde Medical College. Clinical information of breast cancer was obtained via a full clinical examination by specialists. All controls were women who underwent breast cancer screening and were clinically confirmed to be free of breast cancer at the same hospital, and they had a negative history of all forms of cancer in their first-degree relatives. All study participants were genetically unrelated women of Han Chinese descent who were consecutively recruited from the Affiliated Hospital of Chengde Medical College between September 2009 and March 2013.

All study participants were classified into two study groups: the breast cancer group and the cancer-free control group. Overall, 606 patients 54.36 (standard deviation: 12.33) years of mean age were diagnosed with sporadic breast cancer, and the rest 633 participants who had no manifest of cancers formed the age- and ethnicity-matched control group with mean age of 55.15 (standard deviation: 9.38) years.

At enrollment, baseline data on age, family history of cancers, age at menarche and menopausal status were recorded. Moreover, additional data on tumor size (from T1 to T4), tumor grade (from G1 to G3), and lymph node (positive or negative) were exclusively presented for breast cancer patients.

### Selection of polymorphisms

The five DNA repair genes under study were X-ray repair complementing defective repair in Chinese hamster cells 1 gene (XRCC1: rs1799782 and rs25487), XRCC2 gene (rs3218536), XRCC3 gene (rs861539), xeroderma pigmentosum, complementation group A gene (XPA: rs1800975) and APEX nuclease (multifunctional DNA repair enzyme) 1 gene (APEX1: rs1760944 and rs1130409). The selection of these functional polymorphisms was based on their wide evaluation in association with various forms of cancer [9]–[15].

### Genotyping

EDTA blood samples were obtained from all study participants at the time of enrollment. Genomic DNA was isolated from peripheral blood leukocytes by using TIANamp Blood DNA Kit (Tiangen Biotect Co., Beijing, China), and then was stored at −40°C until required for batch genotyping. The polymerase chain reaction-ligase detection reaction (PCR-LDR) method [16] was adopted to determine the genotypes of seven examined polymorphisms in this study.

To discriminate specific bases of each polymorphism, we synthesized two specific probes and one common probe, and labeled the common probe 6-carboxy-fluorescein (FAM) at the 3′ end and phosphorylated at the 5′ end. The multiplex ligation reaction was conducted in a volume of 10 μl containing 2 μl of PCR product, 1 μl of 10×Taq DNA ligase buffer, 1 μM of each discriminating probe, and 5 U of Taq DNA ligase. After ligation reaction, 1 μl of LDR reaction product was mixed with 1 μl of ROX passive reference and 1 μl of loading buffer before being denatured at 95°C for 3 min and chilled rapidly on ice. The fluorescent products of the LDR were differentiated using an ABI 3730XL sequencer (Applied Biosystems, California, USA).

To test the accuracy of the PCR-LDR method, 48 DNA samples were randomly selected and run in duplicates with 100% concordance.

### Statistical analysis

Continuous and categorical variables were compared between breast cancer patients and controls by the unpaired t-test and the χ^2^ test, respectively. A Pearson goodness-of-fit test was conducted to assess the Hardy-Weinberg equilibrium. Binary Logistic regression models were used to evaluate the additive (major homozygotes versus heterozygotes versus minor homozygotes), dominant (major homozygotes versus heterozygotes plus minor homozygotes), and recessive (major homozygotes plus heterozygotes versus minor homozygotes) models of inheritance after controlling for age at enrollment, and risk estimates were expressed as odds ratio (OR) and 95% confidence interval (95% CI). The statistical analyses described above were completed with the SAS software for Windows (version 8.1) (SAS Institute, Cary, North Carolina, USA). Statistical power was estimated by PS (Power and Sample Size Calculations) software (version 3.0.7, Nashville, TN, USA).

Analysis of allele combinations was adopted to examine the joint effect of seven polymorphisms on breast cancer risk, and their frequencies were estimated by the haplo.em program implemented in Haplo.stats software (version 1.4.0, Rochester, MU, USA). The haplo.em program computes the maximum likelihood estimates of allele combination probabilities using the progressive insertion algorithm which progressively inserts batches of loci into the allele combinations of growing lengths. To avoid false-positive results, only allele combination with frequency of over 3% in all study participants was considered in this analysis. P values were calculated based on 1000 simulations.

To explore the potential interactions of multiple polymorphisms of DNA repair genes, a promising data-mining open-source approach multifactor dimensionality reduction (MDR) was employed (version 3.0, available at the website http://www.epistasis.org) [17], [18]. This approach aims to identify the overall best combination of all quantities (from one locus to seven loci). The accuracy of each best model was evaluated by a Bayes classifier in the context of 10-fold cross-validation. A single best model has the maximal testing accuracy and cross-validation consistency simultaneously. The cross-validation consistency is a measure of the number of times of 10 divisions of the dataset that the best model is extracted. Permutation testing corrects for multiple testing by repeating the entire analyses on 1000 datasets that are consistent with the null hypothesis.

## Results

### Baseline characteristics

Details of the study population are shown in Table 1. Age at enrollment did not differed significantly between breast cancer patients and controls (P = 0.205). The percentage of family history of cancers was 10.56% in breast cancer patients. The average age at menarche was 14.60 (standard deviation: 1.63) years. Breast cancer patients with menarche age of 12 years or less, 13–14 years and 15 years or more accounted for 22.44%, 37.13% and 40.34%, respectively. Nearly half of breast cancer patients had postmenopausal status at enrollment (49.83%). 49.80% and 42.54% of patients had tumor size of T1 and T2, and 50.18% and 44.32% patients had tumor grade of G2 and G3, respectively. The percentage of positive lymph node was 42.13% in breast cancer patients.

**Table 1:** The baseline characteristics of all study participants.

Characteristics	Patients (n = 606)	Controls (n = 633)
Age (years)	54.36±12.33	55.15±9.38
Family history of other cancers	10.56%	0%
Menarche age (years)	14.60±1.63	NA
≤12	22.44%	NA
13–14	37.13%	NA
15 +	40.43%	NA
Menopausal status		
Premenopause	50.17%	NA
Postmenopause	49.83%	NA
Tumor size (T1–T4)		
T1	49.80%	NA
T2	42.54%	NA
T3	3.83%	NA
T4	3.83%	NA
Tumor grade (G1–G3)		
G1	4.87%	NA
G2	50.81%	NA
G3	44.32%	NA
Lymph node (+)	42.13%	NA

Data were expressed as mean ± standard deviation unless otherwise indicated.

### Single-locus analysis

Table 2 shows the genotype and allele comparisons of seven polymorphisms under study between patients and controls and their risk prediction for breast cancer under three genetic models of inheritance. No deviation from Hardy-Weinberg equilibrium was noted in controls for all polymorphisms. The genotypes and alleles of XRCC1 gene rs25487 and XPA gene rs1800975 differed significantly between the two groups, even after the Bonferroni correction (Bonferroni significance threshold P = 0.05 divided by the total number of examined polymorphisms (n = 7): P = 0.007). The power to reject the null hypothesis of no differences in mutant allele frequencies of rs25487 and rs1800975 between the two groups was 81.9% and 92.8%, respectively. There was marginal significance in the alleles of XRCC3 gene rs861539 (P = 0.037) and in the genotypes of APEX1 gene rs1130409 (P = 0.026), while no significance was attained after the Bonferroni correction.

**Table 2:** Genotype distributions and allele frequencies of seven polymorphisms under study between patients and controls and their risk prediction for breast cancer under three genetic models of inheritance.

Gene: polymorphism	W/M	Status	WW	WM	MM	M (%)	Three genetic models (OR; 95% CI; P*)	
XRCC1: rs1799782	C/T	Patients	279	263	64	32.26	Additive	0.97; 0.82–1.15; 0.749
		Controls	282	286	65	32.86	Dominant	0.94; 0.75–1.18; 0.598
		P for χ^2^-test	0.819			0.751	Recessive	1.03; 0.72–1.49; 0.866
XRCC1: rs25487	G/A	Patients	318	209	79	30.28	Additive	1.28; 1.07–1.51; 0.006
		Controls	347	254	32	25.12	Dominant	1.1; 0.88–1.37; 0.408
		P for χ^2^-test	<0.0005			0.004	Recessive	2.82; 1.84–4.32; <0.001
XRCC2: rs3218536	G/A	Patients	166	280	160	49.50	Additive	1.11; 0.95–1.29; 0.196
		Controls	184	305	144	46.84	Dominant	1.09; 0.85–1.39; 0.513
		P for χ^2^-test	0.325			0.185	Recessive	1.22; 0.94–1.58; 0.135
XRCC3: rs861539	C/T	Patients	510	91	5	8.33	Additive	1.38; 1.02–1.88; 0.038
		Controls	557	74	2	6.16	Dominant	1.38; 1.0–1.91; 0.052
		P for χ^2^-test	0.104			0.037	Recessive	2.62; 0.51–13.58; 0.25
XPA: rs1800975	A/G	Patients	201	268	137	44.72	Additive	0.77; 0.67–0.9; 0.001
		Controls	157	299	177	51.58	Dominant	0.66; 0.52–0.85; 0.001
		P for χ^2^-test	0.003			0.001	Recessive	0.75; 0.58–0.97; 0.031
APEX1: rs1760944	G/T	Patients	177	293	136	46.62	Additive	1.01; 0.87–1.19; 0.869
		Controls	177	326	130	46.29	Dominant	0.94; 0.74–1.2; 0.628
		P for χ^2^-test	0.520			0.869	Recessive	1.12; 0.85–1.47; 0.414
APEX1: rs1130409	G/T	Patients	389	168	49	21.95	Additive	1.15; 0.95–1.38; 0.144
		Controls	415	190	28	19.43	Dominant	1.06; 0.84–1.34; 0.614
		P for χ^2^-test	0.026			0.122	Recessive	1.9; 1.18–3.07; 0.009

*Abbreviations*: W/M, wild allele/mutant allele; OR, odds ratio; 95% CI, 95% confidence interval. P for χ^2^ test was calculated based on the 3×2 contingency tables for genotype comparisons and on the 2×2 contingency tables for allele comparisons. *Controlling for age at enrollment.

The odds of having breast cancer for rs25487 was significant under both additive (OR = 1.28; 95% CI: 1.07–1.51; P = 0.006) and recessive (OR = 2.82; 95% CI: 1.84–4.32; P<0.001) models, and remained significant after the Bonferroni correction (Table 2). Moreover, the risk of rs1800975 for breast cancer was significantly reduced by 23% (OR = 0.77; 95% CI: 0.67–0.9; P = 0.001) and 34% (OR = 0.66; 95% CI: 0.52–0.85; P = 0.001) under both additive and dominant models, respectively, even with a Bonferroni corrected alpha of 0.05/7.

In addition, given that 10.56% of breast cancer patients (n = 64) had reported a family history of other forms of cancer, further subgroup analysis was conducted by excluding these 64 breast cancer patients in order to eliminate the potential confounding of family history (Table S1). Overall there were no material changes for comparative results and risk estimates of all polymorphisms under study, except for a slight change for rs851539 in XRCC3 gene. The association of this polymorphism with breast cancer was slightly substantiated (P for χ2 test: 0.036 for genotype and 0.010 for allele), while no significance was reached after applying the stringent Bonferroni correction (Bonferroni significance threshold P = 0.05/7).

### Allele combination analysis

The joint effect of seven polymorphisms under study from five DNA repair genes is summarized in Table 3. The most common allele combination was C-G-G-C-G-G-G (alleles in order of rs1799782, rs25487, rs3218536, rs861539, rs1800975, rs1760944 and rs1130409), which was overrepresented in controls (6.66% versus 2.96% in breast cancer patients, P_Sim_ = 0.002, significant at a Bonferroni corrected alpha of 0.05/11). Except for this combination, there was no observable significance for the other allele combinations.

**Table 3:** The allele combinations of seven polymorphisms under study between breast cancer patients and controls.

Allele combination*	All (%)	Patients (%)	Controls (%)	P_Sim_
C-G-G-C-G-G-G	5.32	2.96	6.66	0.002
C-G-A-C-G-T-G	4.19	2.10	4.48	0.026
C-G-G-C-A-G-G	4.14	5.36	2.89	0.625
C-A-G-C-A-G-G	4.05	4.82	3.08	0.172
C-G-G-C-G-T-G	4.04	4.07	4.48	0.022
C-G-A-C-A-T-G	3.94	4.72	4.77	0.757
C-G-A-C-A-G-G	3.90	3.54	3.65	0.965
T-G-G-C-G-G-G	3.34	4.00	4.24	0.137
T-G-A-C-G-G-G	3.27	2.53	2.75	0.327
T-G-A-C-A-T-G	3.27	3.26	3.31	0.894
C-G-G-C-A-T-G	3.13	2.68	3.18	0.797

*Alleles were arranged according to rs1799782, rs25487, rs3218536, rs861539, rs1800975, rs1760944 and rs1130409.

### Interaction analysis

A data-mining analytical approach MDR was adopted to explore the potential interactions of multiple polymorphisms of five DNA repair genes, and the results are summarized in Table 4. Each overall best model of all quantities is weighed by testing accuracy and cross-validation consistency. Overall, the two-locus model including rs1800975 and rs25487 emerged as the best MDR model. This model had the maximal testing accuracy of 0.654 and the maximal cross-validation consistency of 10 out of 10, which was significant at 0.001, indicating that a model this good or better was observed one out of 1000 permutations and thus unlikely hinged on the null hypothesis of null association.

**Table 4:** Summary of multifactor dimensionality reduction (MDR) analysis.

Each overall best model		Testing accuracy	CVC	P
One-locus:	rs1800975	0.621	7	0.070
Two-locus:	One-locus plus rs25487	0.654	10	0.001*
Three-locus:	Two-locus plus rs1760944	0.630	6	0.057
Four-locus:	Three-locus plus rs3218536	0.622	8	0.063
Five-locus:	Four-locus plus rs1799782	0.596	6	0.292
Six-locus:	Five-locus plus rs1130409	0.618	10	0.171
Seven-locus:	All examined polymorphisms	0.612	10	0.180

*Abbreviations*: CVC, cross-validation consistency. *The overall best MDR model.

## Discussion

In this study, we sought to explore the potential interactions of seven common polymorphisms of five DNA repair genes in association with breast cancer among 1239 Han Chinese women. The key finding was that two polymorphisms, XRCC1 gene rs25487 and XPA gene rs1800975, might exert both independent and interactive effects on the development of breast cancer. This study, to the authors' knowledge, is the first report assessing the association of multiple DNA repair genes and polymorphisms, both individually and interactively, with breast cancer risk in Han Chinese women.

In view of the ubiquity of epistasis in determining susceptibility to common human diseases [19], to examine the interactions of multiple genes in common pathologic pathways should be a priority. In such context, MDR has been developed as a promising data-mining approach for overcoming some limitations of traditional parametric statistics such as logistic regression for the detection and characterization of high-order gene-gene and gene-environmental interactions [17], [18]. This approach is nonparametric and model-free in design, and has been successfully applied to detect and characterize high-order gene-gene and gene-environment interactions in studies with relatively small samples [20], [21]. For the present study, application of MDR to breast cancer case-control data set identified a statistically significant two-locus best model from five DNA repair genes. It is not surprising to note that the two polymorphisms in overall best model were strikingly significant in our single-locus analysis, reinforcing the robustness of MDR approach. Moreover, the interactive role of these two polymorphisms was particularly evident in protection against the development of breast cancer, as our allele combination analysis indicated that the estimated frequencies of combinations were consistently higher in controls than patients for those carrying rs25487-G and rs1800975-G alleles, especially for the most common allele combination. Although empirical and theoretical studies have suggested that MDR is a useful method for identifying epistasis, the power of MDR in the presence of noise that is common to many epidemiological studies is unknowable. Furthermore, we cannot exclude the possible existence of residual confounding from the incompletely measured or unmeasured physiologic covariates. Considering the magnitude of risk estimates and the mutual validation of different analytical methods, it seems unlikely that our findings could be explained by confounding.

Although epidemiological studies on DNA repair genes and breast cancer risk have been undertaken extensively across different populations, the results are inconsistent and inconclusive. For example, Roberts et al in Caucasians observed an increased risk of XRCC1 gene rs25487 for breast cancer in postmenopausal women [22], which was consistent with the results of the present study, as well as a recent meta-analysis by Wu et al on 44 independent case-control studies [23]. However, Al Mutairi et al in Saudi patients failed to confirm this association, and instead they found that another polymorphism rs1799782 in XRCC1 gene was associated with the significant risk of breast cancer [24]. Besides the environmental and cultural divergences, it cannot be totally ruled out that the evolutionary history of linkage disequilibrium patterns will vary significantly across different ethnic populations. Generally, a locus is in close linkage with another nearly causal locus in one ethnic group but not in another [25]. As a consequence, there is a need to construct a database of breast cancer-susceptibility genes or polymorphisms in each racial/ethnic group. Also it is of clinical importance to incorporate joint and synergistic analytical strategies for the potential disease-susceptibility genetic defects by scanning DNA repair genes to facilitate the identification of individuals at high risk of developing breast cancer in future clinical screening.

Several limitations of the present study merit consideration. First, this study was conducted on a retrospective case-control design, which has inherent drawbacks and precludes causal inferences [26]. Second, this study of 1239 participants might not be powered enough to address small risk effects. Third, due to our design flaw, some baseline data on age at menarche and menopausal status were not available for controls, as well as other reproducible risk factors for breast cancer, which prevented further adjustment in risk estimates and may have overestimated the true effect size. However, this lack of information is unlikely to affect the validity of our findings, because this study involved homogeneous breast cancer patients and well-matched controls. Fourth, only seven common polymorphisms from five DNA repair genes were evaluated in this study, and it is highly encouraged to incorporate other polymorphisms, especially the low-penetrance polymorphisms of DNA repair genes. Fifth, although MDR is a method to improve the identification of polymorphism combinations associated with disease risk, it is not without drawbacks, such as computational intensiveness, indistinct interpretation, lack of sensitivity, and heterogeneity-free assumption [27], [28]. Last but not the least, because our study sample was entirely of Han Chinese ancestry, we avoided confounding by ethnicity but at the same time, we reduced the generalizability of our findings to other ethnic populations.

In conclusion, our findings provide clear evidence that XRCC1 gene rs25487 and XPA gene rs1800975 might exert both independent and interactive effects on the development of breast cancer. As breast cancer is a multifactorial complex disorder, large well-designed longitudinal studies attempting to account for high-order gene-gene and gene-environment interactions, as well as *in-vitro* and *in-vivo* studies seeking to provide biological or clinical implications of DNA repair genes in susceptibility to breast cancer, are required in future investigation.

## Supporting Information

Table S1 **Genotype distributions and allele frequencies of seven polymorphisms under study between breast cancer patients without a family history of other cancers and controls, as well as their risk prediction for breast cancer under three genetic models of inheritance.**
(DOC)
