# Supplementary material for: The Relationship between Seven Common Polymorphisms from Five DNA Repair Genes and the Risk for Breast Cancer in Northern Chinese Women
Source: PLoS One. 2014 Mar 18;9(3):e92083. doi: 10.1371/journal.pone.0092083 (PMC3958445; doi:10.1371/journal.pone.0092083)
Supplement: Table S1 — Genotype distributions and allele frequencies of seven polymorphisms under study between breast cancer patients without a family history of other cancers and controls, as well as their risk prediction for breast cancer under three genetic models of inheritance. (DOC) [file pone.0092083.s001.doc]

**Supplementary Table S1.** Genotype distributions and allele frequencies of seven polymorphisms under study between breast cancer patients without a family history of other cancers and controls, as well as their risk prediction for breast cancer under three genetic models of inheritance

| **Gene: polymorphism** | **W/M** | **Status** | **WW** | **WM** | **MM** | **M (%)** | **Three genetic models**  **(OR; 95% CI; P^*^)** | |
| --- | --- | --- | --- | --- | --- | --- | --- | --- |
| XRCC1: rs1799782 | C/T | Patients | 248 | 236 | 58 | 32.47 | Additive | 0.98; 0.83-1.17; 0.849 |
|  |  | Controls | 282 | 286 | 65 | 32.86 | Dominant | 0.95; 0.76-1.20; 0.682 |
|  |  | P for χ^2^-test | 0.851 | | | 0.842 | Recessive | 1.05; 0.72-1.52; 0.80 |
| XRCC1: rs25487 | G/A | Patients | 288 | 179 | 75 | 30.35 | Additive | 1.29; 1.08-1.54; 0.004 |
|  |  | Controls | 347 | 254 | 32 | 25.12 | Dominant | 1.08; 0.86-1.36; 0.525 |
|  |  | P for χ^2^-test | <0.0005 | | | 0.005 | Recessive | 3.22; 2.07-5.0; <0.001 |
| XRCC2: rs3218536 | G/A | Patients | 149 | 247 | 146 | 49.72 | Additive | 1.12; 0.95-1.31; 0.176 |
|  |  | Controls | 184 | 305 | 144 | 46.84 | Dominant | 1.08; 0.84-1.39; 0.550 |
|  |  | P for χ^2^-test | 0.252 | | | 0.163 | Recessive | 1.25; 0.96-1.63; 0.097 |
| XRCC3: rs861539 | C/T | Patients | 449 | 89 | 4 | 8.95 | Additive | 1.50; 1.10-2.05; 0.011 |
|  |  | Controls | 557 | 74 | 2 | 6.16 | Dominant | 1.52; 1.09-2.11; 0.013 |
|  |  | P for χ^2^-test | 0.036 | | | 0.010 | Recessive | 2.35; 0.43-12.85; 0.326 |
| XPA: rs1800975 | A/G | Patients | 188 | 221 | 133 | 44.93 | Additive | 0.79; 0.67-0.92; 0.002 |
|  |  | Controls | 157 | 299 | 177 | 51.58 | Dominant | 0.62; 0.48-0.80; <0.001 |
|  |  | P for χ^2^-test | 0.001 | | | 0.001 | Recessive | 0.84; 0.64-1.09; 0.185 |
| APEX1: rs1760944 | G/T | Patients | 154 | 264 | 124 | 47.23 | Additive | 1.05; 0.89-1.23; 0.580 |
|  |  | Controls | 177 | 326 | 130 | 46.29 | Dominant | 0.98; 0.76-1.27; 0.895 |
|  |  | P for χ^2^-test | 0.545 | | | 0.647 | Recessive | 1.17; 0.88-1.54; 0.277 |
| APEX1: rs1130409 | G/T | Patients | 348 | 150 | 44 | 21.96 | Additive | 1.16; 0.96-1.41; 0.114 |
|  |  | Controls | 415 | 190 | 28 | 19.43 | Dominant | 1.07; 0.84-1.36; 0.569 |
|  |  | P for χ^2^-test | 0.028 | | | 0.131 | Recessive | 2.0; 1.23-3.24; 0.005 |

*Abbreviations:* W/M, wild allele/mutant allele; OR, odds ratio; 95% CI, 95% confidence interval. P for χ^2^ test was calculated based on the 3×2 contingency tables for genotype comparisons and on the 2×2 contingency tables for allele comparisons. *Controlling for age at enrollment.
